# Mutagenesis Mapping of the Protein-Protein Interaction Underlying FusB-Type Fusidic Acid Resistance

**DOI:** 10.1128/AAC.00198-13

**Published:** 2013-10

**Authors:** Georgina Cox, Thomas A. Edwards, Alex J. O'Neill

**Affiliations:** Antimicrobial Research Centre and School of Molecular and Cellular Biology, University of Leeds, Leeds, United Kingdom

## Abstract

FusB-type proteins represent the predominant mechanism of resistance to fusidic acid in staphylococci and act by binding to and modulating the function of the drug target (elongation factor G [EF-G]). To gain further insight into this antibiotic resistance mechanism, we sought to identify residues important for the interaction of FusB with EF-G and thereby delineate the binding interface within the FusB–EF-G complex. Replacement with alanine of any one of four conserved residues within the C-terminal domain of FusB (F_156_, K_184_, Y_187_, and F_208_) abrogated the ability of the protein to confer resistance to fusidic acid; the purified mutant proteins also lost the ability to bind S. aureus EF-G *in vitro. E. coli* EF-G, which is not ordinarily able to bind FusB-type proteins, was rendered competent for binding to FusB following deletion of a 3-residue tract (_529_SNP_531_) from domain IV of the protein. This study has identified key regions of both FusB and EF-G that are important for the interaction between the proteins, findings which corroborate our previous *in silico* prediction for the architecture of the complex formed between the resistance protein and the drug target (G. Cox, G. S. Thompson, H. T. Jenkins, F. Peske, A. Savelsbergh, M. V. Rodnina, W. Wintermeyer, S. W. Homans, T. A. Edwards, and A. J. O'Neill, Proc. Natl. Acad. Sci. U. S. A. 109:2102-2107, 2012).

## INTRODUCTION

The antibiotic fusidic acid (FA) is employed for the treatment of superficial and systemic disease caused by staphylococci and remains one of the few oral agents available for treating infections caused by methicillin-resistant Staphylococcus aureus (MRSA) (1). FA inhibits bacterial protein synthesis through interaction with elongation factor G (EF-G) (2, 3), a G protein responsible for catalyzing translocation of peptidyl-tRNA from the A site to the P site of the ribosome (4). Once translocation has occurred, EF-G dissociates from the ribosome, vacating the A site and allowing the next aminoacyl-tRNA species to enter the ribosome. In the presence of FA, the drug binds to EF-G and inhibits its dissociation from the ribosome, thereby preventing further protein synthesis and causing cessation of bacterial growth (2, 5).

Staphylococcal resistance to FA has increased considerably in recent years, threatening the clinical utility of the drug (1, 6–9). The predominant route to FA resistance in clinical strains of S. aureus and other staphylococci involves horizontal acquisition of determinants encoding FusB-type resistance proteins (6, 7, 10). These proteins bind to EF-G and drive its release from the ribosome posttranslocation, even in the presence of FA (11, 12). We recently solved the first structure of a FusB-type protein (FusC) and broadly localized regions of both EF-G and FusB-type proteins that participate in the interaction between the two binding partners (11). The FusC crystal structure revealed a two-domain metalloprotein, the C-terminal domain of which contains a novel 4-cysteine (C4) zinc binding fold (ZBF) that interacts with the C-terminal domains of EF-G (11).

In the present study, we sought to gain further insight into FusB-type proteins and their interaction with EF-G. Specifically, we identified residues in both FusB and EF-G that participate in the formation of the FusB–EF-G complex, thereby permitting more precise delineation of the binding interface between this family of resistance proteins and the drug target.

## MATERIALS AND METHODS

### Expression and purification of recombinant proteins.

The FusB and S. aureus EF-G proteins were expressed and purified as described previously (11, 12). A construct for overexpression of Escherichia coli EF-G was generated by PCR amplification of *fusA* from E. coli JM109 (Promega, Southampton, United Kingdom) and ligation of this amplicon into plasmid pET-29b (Novagen, WI, USA). Deletion of residues _529_SNP_531_ from E. coli EF-G was achieved by PCR amplification and blunt-ended ligation of two DNA fragments of *fusA* flanking this region, followed by ligation into pET-29b. E. coli EF-G was overexpressed and purified as described previously for S. aureus EF-G (11).

### Alanine-scanning mutagenesis of FusB.

Expression of *fusB* in S. aureus from the tetracycline-regulatable expression plasmid pAJ96 was achieved as previously described (10). Site-directed mutagenesis of *fusB* in this construct was performed by using the QuikChange II kit (Agilent Technologies, Cheshire, United Kingdom), according to the manufacturer's guidelines, and employed gel-purified oligonucleotide primers (Eurofins MWG Operon, Ebersberg, Germany). Constructs were propagated in E. coli, followed by electroporation into S. aureus RN4220 (13). MICs of FA were determined by agar dilution in Iso-Sensitest agar, using inocula of 10^6^ CFU per spot. To induce expression of *fusB* from the *xyl/tetO* promoter on pAJ96, cultures were incubated with 250 ng anhydrotetracycline/ml for 3 h at 37°C prior to susceptibility testing.

### *In vitro* protein binding studies.

Analytical gel filtration chromatography was employed for *in vitro* analysis of binding of purified FusB mutant proteins to EF-G, and eluted samples were analyzed by SDS-PAGE (11). For binding studies, purified EF-G (2 mg) was incubated with purified FusB (10 mg) at 4°C in a final volume of 2 ml for 1 h. Samples were applied onto a 16/60 Superdex 75 prep grade prepacked column (GE Healthcare, Buckinghamshire, United Kingdom), eluted in running buffer (20 mM Tris-HCl [pH 8.0], 300 mM NaCl, and 1 mM dithiothreitol [DTT]) at a flow rate of 0.5 ml/min, and analyzed by SDS-PAGE. The gel filtration column was calibrated by using a low-molecular-weight calibration kit (GE Healthcare) according to the manufacturer's guidelines, allowing determination of the molecular mass of eluted proteins. Isothermal titration calorimetry (ITC) was performed as described previously (11).

## RESULTS

### Identification of residues in FusB important for mediating interaction with EF-G.

**Fig 1:**
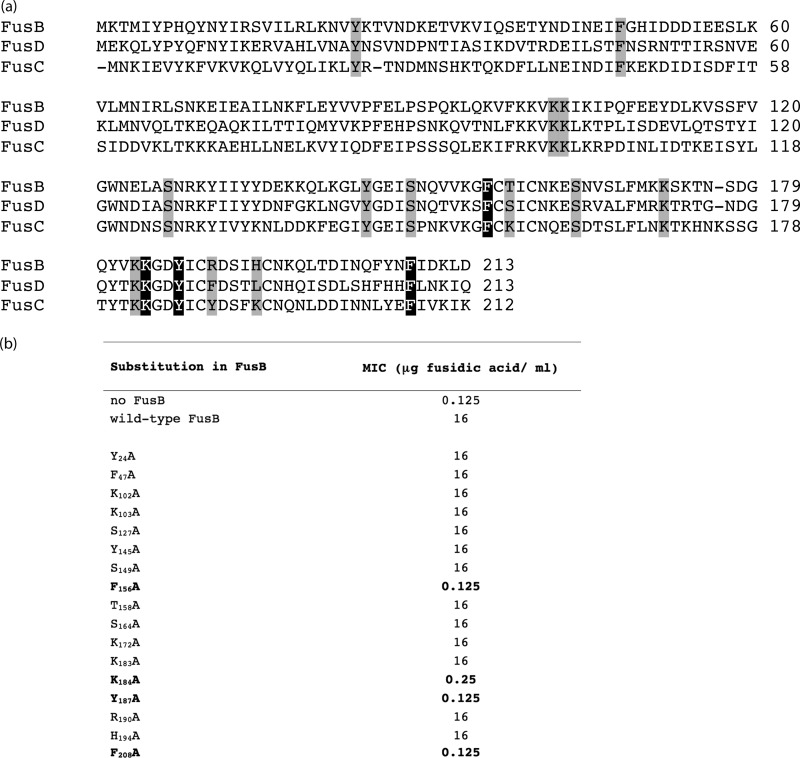
Alanine-scanning mutagenesis of the FusB protein. (a) Sequence alignment of staphylococcal FusB-type proteins (GenBank accession numbers AAL12234 for FusB, YP_042173 for FusC, and YP_302255 for FusD), with residues selected for site-directed mutagenesis highlighted. Residues highlighted in gray are substitutions that did not affect the ability of FusB to protect EF-G, while those highlighted in black correspond to substitutions that abrogated binding of FusB to EF-G. (b) Effect of alanine-scanning mutagenesis of the FusB protein on the FA susceptibility of S. aureus RN4220(pAJ96:*fusB*). Amino acid substitutions leading to reduced resistance to FA are shown in boldface type.

In a previous study, we employed nuclear magnetic resonance (NMR) chemical shift mapping to show that the EF-G binding site of the FusB-type proteins lies within the C-terminal domain of the latter (11). To more precisely define the EF-G binding site, we undertook alanine-scanning mutagenesis (14) of FusB to identify residues essential for the interaction with EF-G. In selecting residues for mutagenesis, we focused on surface-exposed residues conserved throughout the staphylococcal family of FusB-type proteins (FusB, FusC, and FusD) (Fig. 1a), preferentially choosing hydrophobic, aromatic, or charged amino acids for substitution since such residues are commonly found to participate in protein-protein interactions (PPIs) (15). We also selected a small number of residues for substitution that we anticipated would not impact the ability of FusB to bind EF-G, to act as negative controls; these included 3 residues not conserved across the FusB-type protein family and 2 residues lying within the N-terminal domain of FusB (Fig. 1a).

**Fig 2:**
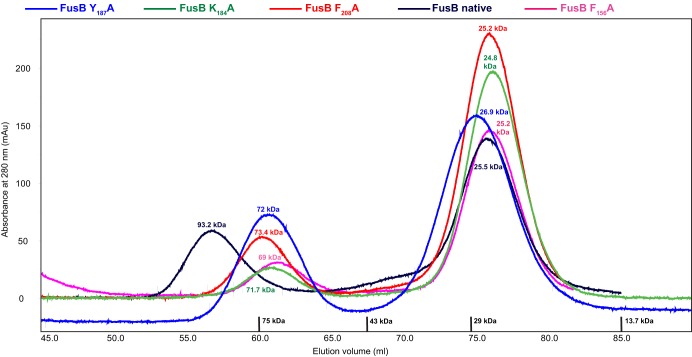
Analytical gel filtration chromatography to examine binding of purified FusB mutant proteins to staphylococcal EF-G. Native FusB (black line) eluted in a complex with EF-G (∼100-kDa complex); the four mutant FusB proteins (Y_187_A, K_184_A, F_208_A, and F_156_A) (∼25 kDa) eluted separately from EF-G (∼75 kDa).

**Fig 3:**
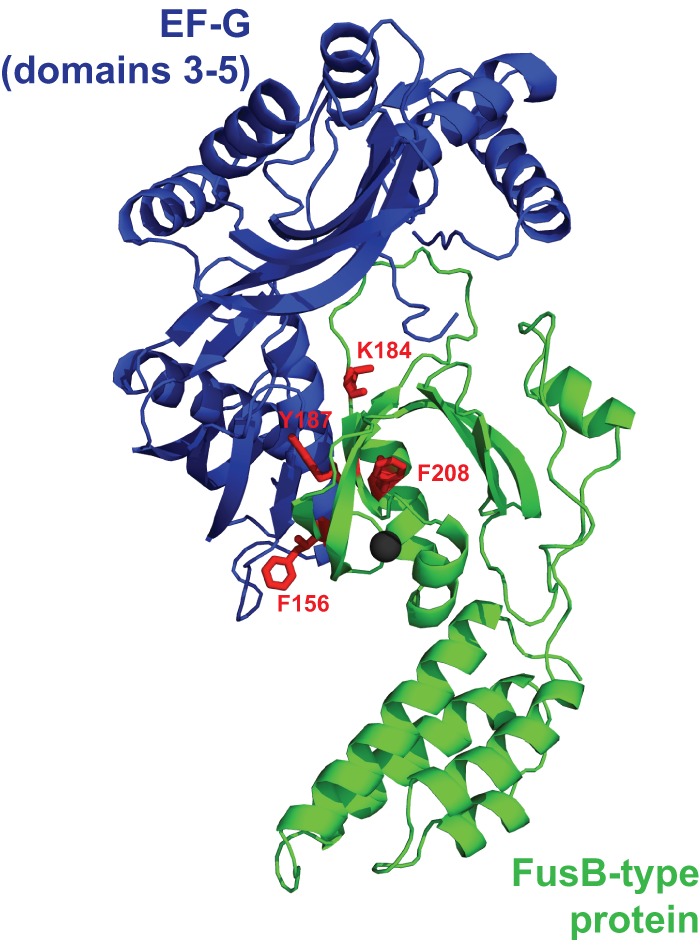
Model of the interaction between FusB-type proteins and EF-G. Residues which reduced the ability of FusB to protect S. aureus from FA and abolished binding of the protein to EF-G (F_156_, K_184_, Y_187_, and F_208_ [labeled and colored red]) are shown mapped onto a model of the FusC–EF-G complex predicted *in silico* (11).

To provide a rapid and direct screen of site-directed FusB mutants for those exhibiting impaired binding to EF-G, we expressed them in S. aureus and used susceptibility testing to identify those mutants exhibiting increased susceptibility to FA compared with a strain expressing unmutagenized FusB. Of 17 amino acids in FusB substituted for alanine (Fig. 1a), 4 (F_156_, K_184_, Y_187_, and F_208_) caused complete, or near-complete, abrogation of the FA resistance phenotype in S. aureus (Fig. 1b). Replacement with alanine of the other conserved and surface-exposed residues (Y_24_, F_47_, K_102_, K_103_, S_127_, Y_145_, S_164_, K_172_, and K_183_), residues not conserved between FusB and FusC (T_158_, R_190_, and H_194_ of FusB), and residues residing within the N-terminal domain of the protein (Y_24_ and F_47_) had no effect on the ability of FusB to protect EF-G against FA *in vivo*. Loss of FA resistance mediated by the four mutant FusB proteins could have resulted from the replacement of residues critical for the interaction with EF-G or by prompting gross changes in the protein that prevented correct folding; the latter seems unlikely since all four mutant proteins could be overexpressed in E. coli and purified in a soluble form. *In vitro* binding studies using analytical gel chromatography established that, in contrast to native FusB, none of the mutant proteins were able to bind purified EF-G (Fig. 2). Mapping of these 4 residues onto our *in silico*-predicted model of a FusB-type protein bound to EF-G (11) revealed that they all reside within the anticipated binding site (Fig. 3).

### Identification of a key region of EF-G responsible for mediating interaction with FusB.

**Fig 4:**
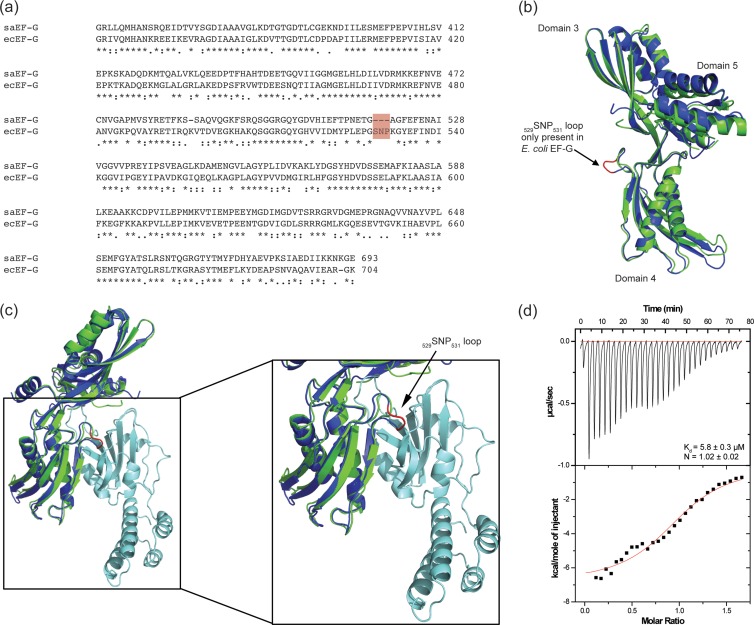
A 3-residue tract (_529_SNP_531_) in domain IV of E. coli EF-G prevents the protein from binding FusB. (a) Sequence alignment of S. aureus EF-G (saEF-G) and E. coli EF-G (ecEF-G) C-terminal domains. The additional 3-residue tract (_529_SNP_531_) present in E. coli EF-G is highlighted in red. (b) Superposition of E. coli EF-G (dark blue) and S. aureus EF-G (green), indicating the location of _529_SNP_531_ in E. coli EF-G (shown in red). (c) Superposition of E. coli EF-G (dark blue) onto our previously reported model of FusC (light blue) and S. aureus EF-G (green) (11), indicating the location of the E. coli EF-G _529_SNP_531_ loop within the FusC–EF-G binding interface. (d) Analysis of binding of E. coli EF-G (Δ_529_SNP_531_) to FusB by ITC. The dissociation constant (*K_d_*) and stoichiometry of the complex (*N*) are indicated.

It was previously established that FusB-type proteins interact with the C-terminal domains (domains III to V) of EF-G (11, 16); however, the precise location of the binding site within this 35-kDa fragment is unclear. To allow us to define key determinants of this interaction on EF-G, we sought to understand the molecular basis for the observation that E. coli EF-G, although exhibiting a high degree of amino acid sequence identity with S. aureus EF-G (∼60%), is unable to bind to FusB-type proteins (11, 12). Comparison of the amino acid sequences of domains III to V of EF-G proteins from S. aureus (GenBank accession number ABD29677.1) and E. coli (GenBank accession number BAE77951.1) revealed numerous small differences between the two proteins (Fig. 4a), including an additional 3-residue tract (_529_SNP_531_) in domain IV of E. coli EF-G (Fig. 4a and b) that is absent from the staphylococcal protein. Overlaying E. coli EF-G onto our *in silico*-generated model of a FusB-type protein bound to S. aureus EF-G (Fig. 4b) revealed that these residues form a short loop lying within the predicted binding interface (Fig. 4c), which could potentially act to prevent interaction of FusB-type proteins with E. coli EF-G through steric hindrance. To investigate this possibility, we deleted _529_SNP_531_ from E. coli EF-G and evaluated the ability of the purified mutant protein to bind FusB. Analytical gel filtration chromatography demonstrated that deletion of _529_SNP_531_ rendered E. coli EF-G capable of binding FusB *in vitro* (data not shown), a finding that we subsequently confirmed using ITC (Fig. 4d).

## DISCUSSION

Since binding of FusB-type proteins to EF-G is central to FA resistance (11, 12), detailed knowledge of the interaction occurring between these proteins will be essential for gaining a more complete understanding of the resistance mechanism. In this study, we sought to map more precisely the binding interface between FusB and EF-G by delineating key residues that impact binding.

Four amino acids in FusB were identified that, when substituted for alanine, abrogated binding of the protein to EF-G, both *in vivo* and *in vitro* (Fig. 1 and 3). All four of these residues lie within the C-terminal domain of FusB, in close proximity to the ZBF (Fig. 3), the region of the protein that we have previously predicted by *in silico* modeling to include the EF-G binding site (11). The majority of these amino acids possess bulky hydrophobic side chains, which may suggest that binding of FusB-type proteins to EF-G is driven by burial of surface-exposed hydrophobic residues. We were intrigued to find that although the K_184_A substitution abrogated the ability of FusB to bind EF-G and mediate FA resistance, substitution of the adjacent residue K_183_ did not. An explanation for this observation is provided by our *in silico* FusC–EF-G model (11), in which K_183_ is orientated away from EF-G and is not therefore anticipated to participate in the interaction between FusB-type proteins and EF-G.

We have previously localized the site of binding of FusB-type proteins on EF-G to a region residing within domains III to V of EF-G (11). Binding studies using FusB and hybrid E. coli-S. aureus EF-G proteins have further emphasized the importance of domain IV of EF-G in the interaction with FusB-type proteins (16). Here we have established that a 3-residue tract (_529_SNP_531_) located in domain IV of E. coli EF-G, but which is absent from S. aureus EF-G, is responsible for preventing binding of FusB-type proteins to the former; deletion of this tract renders E. coli EF-G competent for binding to FusB *in vitro*. These 3 residues form a short loop in E. coli EF-G that lies close to the *in silico*-predicted binding site of FusB-type proteins (11) (Fig. 4c) and likely causes steric occlusion of FusB-type proteins from E. coli EF-G. This observation indicates that FusB-type proteins are in direct contact with, or in very close proximity to, domain IV of EF-G in the vicinity of _529_SNP_531_. Since this region of EF-G makes direct contact with the ribosome (17), this observation supports our proposal that binding of FusB-type proteins to EF-G would prevent EF-G from making normal ribosomal contacts and that binding of EF-G to FusB-type proteins and to the ribosome are mutually exclusive events (11). We note that although deletion of _529_SNP_531_ from E. coli EF-G is sufficient to allow the protein to bind FusB, the affinity of this interaction (*K_d_* [dissociation constant] of 5.8 μM) (Fig. 4d) is considerably lower than that observed for FusB with S. aureus EF-G (*K_d_* of 59 nM) (11), indicating that other amino acid differences between S. aureus and E. coli EF-G proteins must also influence the interaction with FusB.

This study has delineated key residues within the family of FusB-type proteins responsible for binding to EF-G and thereby mediating resistance to FA. Given that the binding of FusB to EF-G can be completely abrogated by substitution of a single amino acid in the former, it is tempting to speculate that the clinical activity of FA could be rejuvenated by the identification of small-molecule inhibitors capable of blocking the PPI between FusB-type resistance proteins and the drug target.
